# Exploration of the radiosensitivity-related prognostic risk signature in patients with glioma: evidence from microarray data

**DOI:** 10.1186/s12967-023-04388-w

**Published:** 2023-09-12

**Authors:** Xiaonan Zhang, Qiannan Ren, Zhiyong Li, Xiaolin Xia, Wan Zhang, Yue Qin, Dehua Wu, Chen Ren

**Affiliations:** 1 Department of Radiation Oncology, Nanfang Hospital of Southern Medical University, Guangzhou, China; 2 Department of Neurosurgery, Nanfang Hospital of Southern Medical University, Guangzhou, China; 3 Department of Radiation Oncology, Yunfu People’s Hospital, Yunfu, Guangdong China

**Keywords:** Glioma, Radiotherapy, Radio-resistance

## Abstract

**Background:**

Gene expression signatures can be used as prognostic biomarkers in various types of cancers. We aim to develop a gene signature for predicting the response to radiotherapy in glioma patients.

**Methods:**

Radio-sensitive and radio-resistant glioma cell lines (M059J and M059K) were subjected to microarray analysis to screen for differentially expressed mRNAs. Additionally, we obtained 169 glioblastomas (GBM) samples and 5 normal samples from The Cancer Genome Atlas (TCGA) database, as well as 80 GBM samples and 4 normal samples from the GSE7696 set. The “DESeq2” R package was employed to identify differentially expressed genes (DEGs) between the normal brain samples and GBM samples. Combining the prognostic-related molecules identified from the TCGA, we developed a radiosensitivity-related prognostic risk signature (RRPRS) in the training set, which includes 152 patients with glioblastoma. Subsequently, we validated the reliability of the RRPRS in a validation set containing 616 patients with glioma from the TCGA database, as well as an internal validation set consisting of 31 glioblastoma patients from the Nanfang Hospital, Southern Medical University.

**Results:**

Based on the microarray and LASSO COX regression analysis, we developed a nine-gene radiosensitivity-related prognostic risk signature. Patients with glioma were divided into high- or low-risk groups based on the median risk score. The Kaplan–Meier survival analysis showed that the progression-free survival (PFS) of the high-risk group was significantly shorter. The signature accurately predicted PFS as assessed by time-dependent receiver operating characteristic curve (ROC) analyses. Stratified analysis demonstrated that the signature is specific to predict the outcome of patients who were treated using radiotherapy. Univariate and multivariate Cox regression analysis revealed that the predictor was an independent predictor for the prognosis of patients with glioma. The prognostic nomograms accompanied by calibration curves displayed the 1-, 2-, and 3-year PFS and OS in patients with glioma.

**Conclusion:**

Our study established a new nine-gene radiosensitivity-related prognostic risk signature that can predict the prognosis of patients with glioma who received radiotherapy. The nomogram showed great potential to predict the prognosis of patients with glioma treated using radiotherapy.

**Supplementary Information:**

The online version contains supplementary material available at 10.1186/s12967-023-04388-w.

## Introduction

Glioma is the most common primary central nervous system (CNS) cancer, accounting for 78.3% of all malignant brain tumors [1–3]. Highly malignant gliomas have a poor prognosis, with a median PFS of less than 7 months [4, 5]. At present, the comprehensive glioma treatment strategy is based on maximal surgical resection, chemotherapy, and radiotherapy. Among them, radiotherapy has significant clinical benefits in improving outcomes for patients with glioma [6, 7]. However, despite its efficacy, 95% of patients with glioblastoma relapse after radiotherapy, with more than 80% of the relapse pattern occurring within the irradiation field [8, 9]. This suggests that radiation resistance of glioma cells may be a significant factor. Several studies have identified potential mechanisms of radio-resistance in gliomas, including glioma cell stemness, DNA damage repair, cell cycle blockade, changes to the tumor microenvironment, and regulation of autophagy [10, 11]. However, the underlying molecular mechanisms of radio-resistance remain unclear. Molecular markers, including 1p/19q co-deletion and mutations in TERT (encoding telomerase reverse transcriptase), P53, and ATRX (encoding alpha thalassemia/mental retardation syndrome X-linked chromatin remodeler), have been identified as crucial predictors of patient prognosis and treatment sensitivity [12–14]. Identifying biomarkers associated with radiotherapy response is essential to understanding the mechanism of glioma radio-resistance.

Some studies [15–17] have demonstrated the prognostic ability of gene signatures for disease prognosis, and the prediction of metastasis and recurrence in patients. And, there are also several studies concentrating on the sensitivity of radiotherapy in patients with gliomas. Lin et al. [18] developed a three-lncRNA signature to predict clinical outcomes in low-grade glioma patients after radiotherapy, which was established according to radiotherapeutic response (complete or partial responses) and divided patients into the radiotherapy-resistant group and radiotherapy-sensitive group and analyzed the differentially expressed lncRNAs. Zhang et al. [19] established a five-microRNA signature according to the association of the LGG (Low Grade Glioma) patients’ overall survival (OS) with microRNA expression. Yan et al. [20] analyzed those genes significantly associated with OS in radiotherapy patients and intersected immune-related genes to construct a 21-gene signature to identify patients with LGGs who would benefit from radiotherapy. However, there are several limitations to those studies. Firstly, most patients underwent surgical resection, and there were no observable lesions to evaluate the radiotherapy response. Second, there are many confounding factors affecting the clinical survival data, including the extent of surgical resection and chemotherapy response. It might lead to clinical response or survival data that cannot fully reflect radio-sensitivity. To address these limitations, we constructed a pair of radio-resistant and radio-sensitive cell lines in vitro, generated a microarray to obtain differentially expressed genes (DEGs) related to radiosensitivity, and combined these DEGs with PFS-related genes obtained from the TCGA database to identify radiosensitivity-related genes. In this study, we sought to identify biomarkers of radiosensitivity in patients with glioma to predict their response to radiotherapy and their PFS. We derived the M059J and M059K cell lines from two parts of the same tumor, with the former being radio-sensitive and the latter being relatively resistant, demonstrating a sensitivity difference of approximately 30-fold. Using microarray analysis, we compared the mRNA expression profiles of the two cell lines and identified several hub genes related to radiosensitivity, constructing a radiosensitivity-related prognostic risk signature (RRPRS). We evaluated the performance of the RRPRS in different cohorts, examined its predictive power in patients with glioma, and developed nomograms to guide clinical practice and predict PFS and OS in glioma patients.

## Methods

### Cell lines and culture

The human glioblastoma cell line M059K (CRL-2365) and M059J (CRL-2366) cells were obtained from the American Type Culture Collection (ATCC, Manassas, VA). M059K cells express normal levels of DNA-dependent protein kinase while M059J cells lack DNA-dependent protein kinase activity. M059K was radio-resistant and M059J was relatively radio-sensitive (the sensitivity of radiotherapy differed by a factor of approximately 30)[21]. M059K and M059J cells were maintained in Dulbecco’s modified Eagle’s medium (DMEM) and Ham’s F12 medium (1:1 mixture) (GIBCO, Australia) supplemented with 10% fetal calf serum (GIBCO), 1 mM non-essential amino acid (Sigma) [22].

### Microarrays and computational analysis

Microarray analysis was performed using the two human glioma cell line M059K and M059J cell as experimental models to select differentially expressed mRNAs. Three independent experiments were conducted for each cell line. Total cellular RNA was extracted with TRIzol Reagent (Invitrogen, Carlsbad, CA) and purified using a RNeasy Mini Kit (Qiagen, Valencia, CA). Subsequently, complementary DNA (cDNA) was synthesized and labeled for microarray hybridization using Arraystar (Rockville, MD). The microarray slides were cleaned and scanned using an Agilent DNA Microarray Scanner (Agilent, Santa Clara, CA). The obtained data were subjected to complete sequencing analysis by KangChen Bio-tech (Project Code: H1211196), and the data were normalized using GeneSpring GX v11.5 software package (Agile Technologies) with low-intensity filtering. To identify statistically significant differentially expressed mRNAs, we applied a Volcano Plot filtering between the two groups based on fold change (> 2.0) and a P-value threshold (< 0.05). The results were further analyzed using hierarchical clustering with the Cluster Treeview software (Stanford University, CA, USA).

### Data collection and preprocessing

GSE7696 is a dataset from the Gene Expression Omnibus (GEO) that explores resistance factors to concurrent chemoradiotherapy and adjuvant chemotherapy, including 80 glioblastoma samples and 4 normal brain tissue samples. The RNA-seq data of those samples were downloaded from the GEO database (https://www.ncbi.nlm.nih.gov/geo/). We used “GEO2R” to analyze differentially expressed genes between tumor tissue and normal tissue. The RNA-sequencing (RNA-seq) data of GBM (glioblastoma) (n = 174), lower grade glioma and glioblastoma (GBMLGG) (n = 702), and normal brain samples (n = 5) were downloaded from The Cancer Genome Atlas dataset (TCGA, https://portal.gdc. cancer.gov) using UCSC Xena (https://xenabrowser.net/datapages/) in August 2022. We excluded some patients who lacked clinical information (such as radiation therapy information) and final included 616 glioma patients, and 152 glioblastomas, respectively. These data were uniformly transformed into FPKM (Fragments Per Kilobase per Million) by the Toil process for comparative analysis. The detailed clinicopathological characteristics of the patients are shown in Table 1. Messenger RNA microarray data from the Chinese Glioma Genome Atlas (CGGA) of diffuse gliomas including clinical information were downloaded from the CGGA website on July, 17, 2022. Patients without radiotherapy information were excluded and a total of 501 patients were enrolled in the CGGA cohort for analysis.

**Table 1:** Demographics and clinicopathological features of patients with glioma in the TCGA and SMU-NFH cohorts

Variables	Training set			Validation set			SMU–NFH set		
	Total (n = 152)	High–risk group (n = 76)	Low–risk group (n = 76)	Total (n = 616)	High–risk group (n = 308)	Low–risk group (n = 308)	Total (n = 31)	High–risk group (n = 16)	Low–risk group (n = 15)
Age(years)		60.1 ± 12.4	59.6 ± 14.8		52.4 ± 14.8	41.2 ± 12.7		43.8 ± 8.1	44.2 ± 16.4
Sex									
Female	54	27	27	267	129	138	14	6	8
Male	98	49	49	349	179	170	17	10	7
KPS									
< 80	31	12	19	54	42	12	11	3	8
≥ 80	85	49	36	136	89	47	20	13	7
NA	36	15	21	426	177	249	–	–	–
Progression									
NO	32	9	23	295	109	186	10	4	6
Yes	120	67	53	321	199	122	21	12	9
OS status									
Alive	64	26	38	370	139	231	15	8	7
Dead	86	50	36	246	169	77	16	8	8
IDH mutation status									
Mutant	10	1	9	388	112	276	7	2	5
Wildtype	138	72	66	219	187	32	24	14	10
NA	4	3	1	9	9	0	–	–	–
MGMT promoter status									
Methylated	52	24	28	NA	NA	NA	3	0	3
Unmethylated	67	36	31	NA	NA	NA	28	16	12
NA	33	16	17	NA	NA	NA	–	–	–
Radiotherapy									
No	39	19	20	190	95	95	10	5	5
Yes	111	57	54	426	213	213	21	11	10
NA	2	0	2	–	–	–	–	–	–
1p/19q codeletion status									
Codeletion	0	0	0	149	68	81	1	1	0
Non–codeletion	147	76	71	462	237	225	30	15	15
NA	5	0	5	5	3	2	–	–	–
Histology									
Glioblastoma	152	76	76	159	139	20	31	16	15
Astrocytoma	–	–	–	164	56	108	–	–	–
Oligoastrocytoma	–	–	–	125	35	90	–	–	–
Oligodendroglioma	–	–	–	168	78	90	–	–	–
WHO Grade									
Grade II	–	–	–	226	73	153	–	–	–
Grade III	–	–	–	231	96	135	–	–	–
Grade IV	152	76	76	159	139	20	31	16	15

*GBM* glioblastoma, *KPS* Karnofsky performance score, *OS status* overall survival status, *NA* not available

### Identification of candidate genes

The “GEO2R” analysis was used to identify differentially expressed genes in 80 glioblastoma samples and 4 normal brain samples from GSE7696. P < 0.05, |log2 foldchange (FC)|> 1 were set as cut-off values. Then, we used the “DESeq2” package in the R software to identify differentially expressed genes in 169 GBM samples and 5 adjacent non-tumor samples. P < 0.05, |log2 foldchange (FC)|> 2 were set as cut-off values. To investigate the response of gliomas to radiotherapy, we identified PFS-associated genes in TCGA using the “survminer” package with a filtering criterion of P < 0.05 and Hazard Ratio (HR) > 1. The above genes that intersected with the DEGs screened by the microarray analysis were identified as candidate genes for the subsequent construction of the radiosensitivity-related prognostic model.

### Gene set enrichment analysis (GSEA)

To explore the different molecular patterns between radioresistant and radiosensitive cell strains, GSEA was performed using MSigDB collections using the “clusterProfiler” package in R [23].

### Establishment of radiosensitivity-related prognostic risk signature (RRPRS)

Then, Lasso COX regression was performed to determine the contributions of radiosensitivity-related genes in survival prediction using the R package “glmnet”, and nine hub genes were used to construct the prognostic risk models [24]. The risk score was calculated according to the standardized TCGA-GBM mRNA expression data in the training set.

$$\mathrm{Risk score }={\sum }_{i}^{n}{x}_{i}{y}_{i}$$

X represents the coefficient of radiosensitivity-related genes in LASSO Cox regression analysis, Y represents the gene expression of radiosensitivity-related genes. By using ten-fold cross-validation, the results of LASSO model were validated. The cross-validation allows us to pick the RRPRS with the λ value having the lowest mean squared error or model variance. We calculated each patient’s risk score and the median risk score was used as the cutoff value to divide patients into high and low-risk subgroups, and the progression-free survival between these two groups was analyzed. The receiver operating characteristic (ROC) curves were produced by the timeROC package to evaluate the prognostic efficiency of the RRPRS. To make the model more convincing, we validated it in the TCGA-GBMLGG cohort and the SMU-NFH cohort. We normalized the expression of relevant genes and calculated the risk score according to the above formula. The patients were also divided into high-risk and low-risk groups based on the median risk score, and compare the PFS between the two groups. The expression of each gene in the RRPRS was also standardized, and the PFS between the two groups was also compared. The nomogram and calibration plots produced for the model were constructed using the rms package (version 6.2) and survival package (version 3.2-10) in R.

### Clinical specimens and study design

From February 2017 to August 2021, a total of 31 patients with glioblastoma were included at the Nanfang Hospital, Southern Medical University (SMU-NFH) cohort. All cases were pathologically diagnosed to be glioblastoma by experienced pathologists in the hospital. None of the patients had received any anti-tumor therapy before tissue biopsy. All patients underwent staging magnetic resonance imaging (MRI) or computed tomography (CT) examination. Tumor assessments were performed at least 6 months using contrast-enhanced CT or MRI. Two radiologists (W-DH and RC) separately assessed all the imaging data, and disagreements were resolved by consensus. This study adhered to ethical guidelines and was approved by the institutional ethical review boards of Southern Medical University Affiliated Nanfang Hospital (Project number: NFEC-202302-081), and the requirement for informed consent was waived. This study has been registered with the Medical Research Registration and Filing Information System (Number: MR-44-23-008141). Supplementary Table S2 contains the demographic and follow-up data of the 31 patients.

### Sample extraction and sequencing

Samples from all 31 patients included in the Southern Medical University cohort were analyzed using RNA-seq. The formalin-fixed, paraffin-embedded (FFPE) tissue were collected before chemotherapy and radiotherapy treatment. RNA Sequencing and analysis were performed at the Genomics Laboratory of GenomicCare Biotechnology (Shanghai, China). For FFPE tissue, RNA was purified using a MagMAX FFPE DNA/RNA Ultra kit (cat# A31881, ThermoFisher), reverse-transcribed using the NEBNext RNA First Strand Synthesis Module (cat# E7525S, NEB, Ipswich, MA, USA), and NEBNext Ultra II non-directional RNA Second Strand Synthesis Module (cat# E6111S, NEB) for cDNA Synthesis. RNA-seq libraries were prepared from the cDNA using SureSelect XT HS and Low Input Library Preparation Kit for ILM (Pre PCR) (cat# G9704, Agilent, Santa Clara, CA, USA) following the manufacturer’s instructions. The libraries were sequenced on an Illumina NovaSeq-6000 Sequencing System (Illumina Inc., San Diego, CA, USA) to generate 150 × 150 paired-end reads. The expression level of each mRNA was presented as fragments per kilobase of transcript per million mapped reads (FPKM).

### Data quality control

RNA-seq reads were mapped to NCBI human genome reference assembly hg19 using STAR (version 020201) [25] and a count matrix was generated. The raw read counts were further normalized by log2-counts per million normalization and the list of genes expressed differentially between groups was generated using DESeq2 [26].

### Statistical analysis

Data analysis was carried out by R software (verion3.6.3) and corresponding R packages [27]. Log-rank tests or Cox regression analysis were used to evaluate the statistical significance for PFS and OS. Receiver operating characteristics (ROC) curves were constructed to examine the sensitivity and specificity of survival prediction. The area under the ROC curve (AUC) values were used to evaluate model performances (using the R package ROCR). Univariate and multivariate Cox regression analyses were used to evaluate the influence of various potential risk factors on PFS in patients with glioma [28]. A significant difference was defined by a P < 0.05.

## Results

### Flow-chart and mRNA expression profile in two human glioma cell lines

**Fig. 1:**
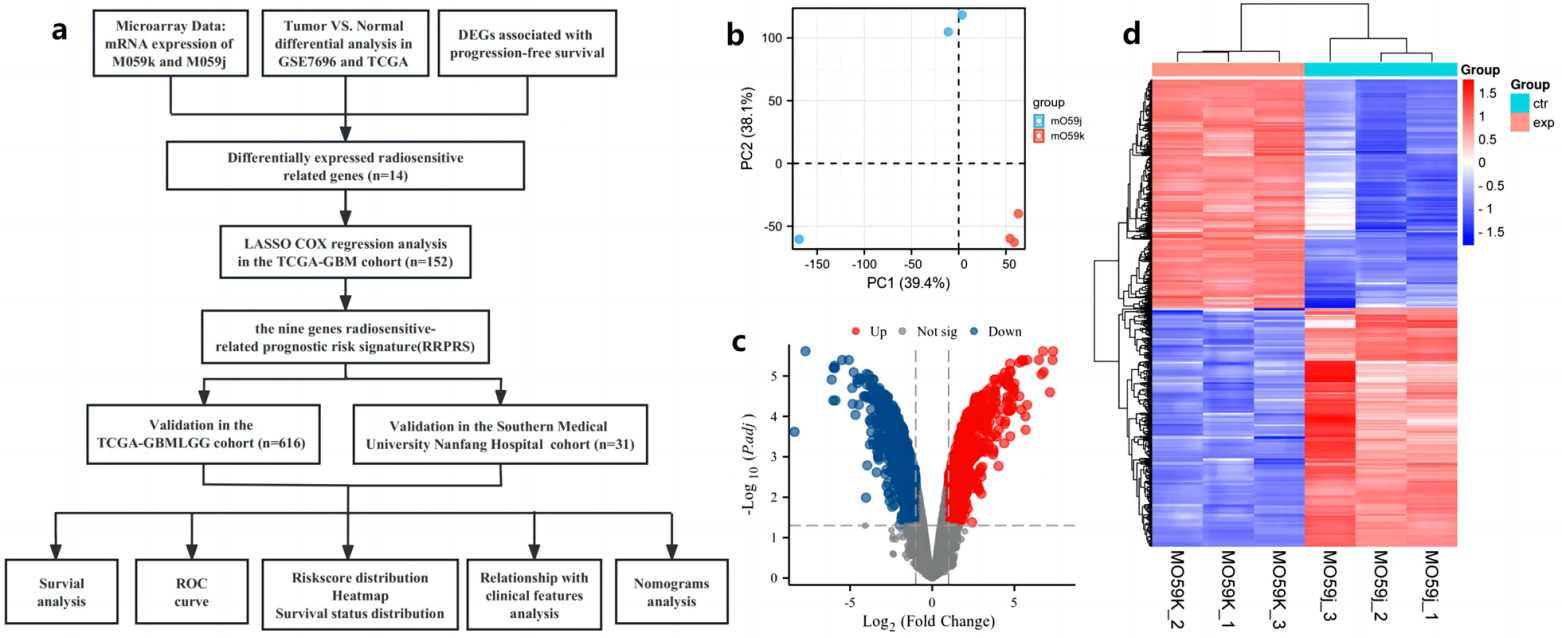
Flow-chart and mRNA expression profile in 2 human glioma cell lines. **a** the workflow of the current study. **b** Principal component analysis (PCA). PCA was performed across the 6 samples (M059K and M059J cells) using normalized log2 gene expression levels. Based on two principal components (PC1 and PC2), M059J (blue data points) and M059K (red data points) showed evidence of separation in gene-expression space. Volcano plots: **c** the vertical lines correspond to 2.0-fold up and down and the horizontal line represents a p-value of 0.05. Thus, the red point in the plot represents the differentially expressed genes with statistical significance; **d** hierarchical clustering shows a distinguishable mRNA expression profiling among samples

We first presented the workflow process of our study in a flow chart (Fig. 1a**)**. To investigate the potential role of genes in the radiosensitivity of glioma, we performed microarray analysis to assess the differentially expressed genes (DEGs) between the M059K cell line and its parental cell line, M059J. The Principal Component Analysis (PCA) of the original dataset between the two cell lines showed a distinct pattern of molecular characterization (Fig. 1b). A volcano plot (Fig. 1c) showed the DEGs between the two groups, and we identified 1850 DEGs, of which 897 were upregulated and 953 were downregulated. (R: ggplot2 package, P < 0.05 and fold change > 2.0). Hierarchical clustering of DEGs between M059K and M059J cells is presented as a heatmap (Fig. 1d). The Gene set enrichment analysis (GSEA) indicated that the radio-resistant cell line M059K was associated with enrichment of angiogenesis, mesenchymal transformation, and interferon response (IFNα), which provided a foundation for our investigation of the mechanism of radio-resistance in patients with glioma (Additional file 1: Fig. S1 a–c).

### Establishment of a radiosensitivity-related prognostic risk signature (RRPRS)

**Fig. 2:**
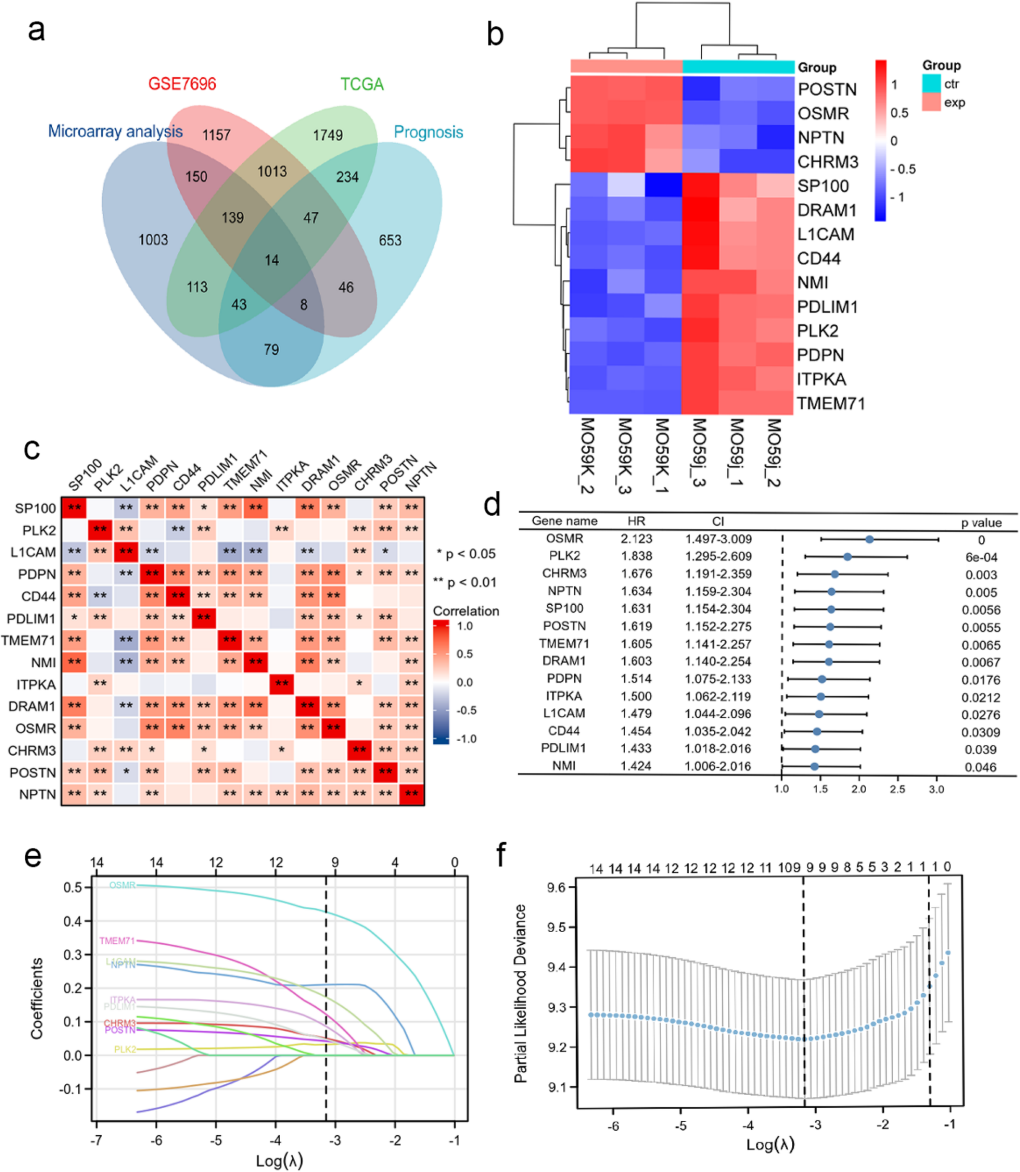
Establishment of a Radiosensitivity-Related Prognostic Risk Signature (RRPRS). **a** venn diagram showing overlapping mRNAs from the results of mRNA microarray analysis (the mRNA microarray analysis screened out 1850 DEGs, after de-duplicating, there were 1549 left), GSE7696 database, and TCGA database prediction (Prognosis is the 1124 genes related to PFS); **b** hierarchical clustering of the six samples with the 14 differentially expressed mRNAs using Euclidean distance and average linkage clustering. Each row represents an individual gene, and each column represents an individual sample; **c** a heatmap of the correlations among multiple genes; **d** Univariate Cox regression analysis of 14 significant DEGs for PFS in the TCGA cohort shown as a forest plot; **e**, **f** Lasso Cox regression analysis of radiosensitivity-related genes

First, we identified 3393 genes that were differentially expressed between 80 glioblastoma samples and four normal brain tissue samples from GSE7696 using “GEO2R”, which was designed to study the factors influencing radiation resistance in glioblastoma. P < 0.05, |log2 foldchange (FC)|> 1 were set as cut-off values. We obtained 169 GBM samples and 5 adjacent non-tumor tissues from TCGA. A total of 3352 differentially expressed genes were identified using the “DESeq2” package with a minimum of twofold change, and a P-value of P < 0.05. For prognostic analysis, Cox proportional hazards analysis was performed using the “survminer” package in R. We identified 1124 genes related to progress-free survival (PFS) based on a filtering criterion of P < 0.05 and hazard ratio (HR) > 1 in the TCGA-GBM data. We intersected the DEGs obtained by microarray analysis, GSE7696, and TCGA and ultimately identified 14 radiosensitivity-related genes. (Fig. 2a). Next, we evaluated the expression levels of these 14 hub genes in the two cell lines (Fig. 2b), and found that some genes were highly expressed in radio-resistant cells while others were highly expressed in radio-sensitive cells. In other words, these 14 key genes had different expression patterns in the two cell lines with different radiotherapy sensitivities. We then created a heat map displaying the cross-correlations between all genes (Fig. 2c), and a forest plot was generated to show the effect of the hub genes on PFS prognosis (Fig. 2d), suggesting that these molecules might have a relationship with the efficacy of radiotherapy in patients with glioma. Lastly, we performed a LASSO Cox regression analysis of the hub genes, which further narrowed the number of these radiosensitivity-related genes to nine, and established an RRPRS (Fig. 2e, f).

### The predictive value of the RRPRS for the PFS of patients with glioma

After screening those patients who lacked radiation therapy information, 152 patients with glioblastoma and 616 patients with glioma in the TCGA database were included in training cohorts and validation cohorts, respectively, while the test cohort consisted of 31 glioblastoma patients from Nanfang Hospital, Southern Medical University (SMU-NFH). The detailed clinicopathological characteristics of the patients are shown in Table 1. Univariate Cox regression analysis showed that age, *IDH* (encoding isocitrate dehydrogenase) mutation status, 1p19q codeletion status, O6-methylguanine (O6-MeG)-DNA methyltransferase (MGMT) status, and RRPRS risk scores correlated significantly with the PFS of patients with glioma (Table 2). Multivariate Cox regression analysis confirmed that RRPRS remained an independent prognostic factor after adjusting for other clinicopathological factors. The same results were also recapitulated in the CGGA database (Additional file 1: Table S1).

**Table 2:** Univariate and multivariate Cox regression analysis of the RRPRS for PFS in patients with glioma

For PFS variables	Training set				Validation set			
	Univariate analysis		Multivariate analysis		Univariate analysis		Multivariate analysis	
	HR (95% CI)	P- value	HR (95% CI)	P- value	HR (95% CI)	P -value	HR (95% CI)	P- value
Sex (male vs female)	1.160 (0.794–1.696)	0.443	0.919 (0.513–1.645)	0.777	1.085 (0.869–1.355)	0.47	1.060 (0.840–1.338)	0.623
Age	1.014 (1.000–1.027)	**0.049**	1.009 (0.985–1.034)	0.47	1.038 (1.030–1.046)	** < 0.001**	1.011 (1.001–1.020)	**0.028**
KPS (≥ 80 vs < 80)	1.478 (0.889–2.457)	0.132	1.215 (0.674–2.192)	0.517	–	–	–	–
1p19q codeletion status (codel vs non-codel)	–	–	–	–	0.29 (0.206–0.408)	** < 0.001**	0.374 (0.233–0.600)	** < 0.001**
IDH mutation status (mutant vs wildtype)	0.323 (0.131–0.798)	**0.014**	0.607 (0.111–3.322)	0.565	0.153 (0.120–0.195)	** < 0.001**	0.488 (0.326–0.731)	** < 0.001**
MGMT status (methylated vs un-methylated)	0.530 (0.341–0.822)	**0.005**	0.974 (0.516–1.839)	0.936	–	–	–	–
Radiotherapy	0.990 (0.965–1.017)	0.481	0.989 (0.962–1.017)	0.439	0.682 (0.527–0.882)	**0.004**	1.601 (1.173–2.186)	**0.003**
RRPRS (high-risk vs low-risk)	3.412 (2.324–5.008)	** < 0.001**	3.605 (1.510–8.611)	**0.004**	2.911 (2.437–3.476)	** < 0.001**	1.878 (1.343–2.625)	** < 0.001**

**Fig. 3:**
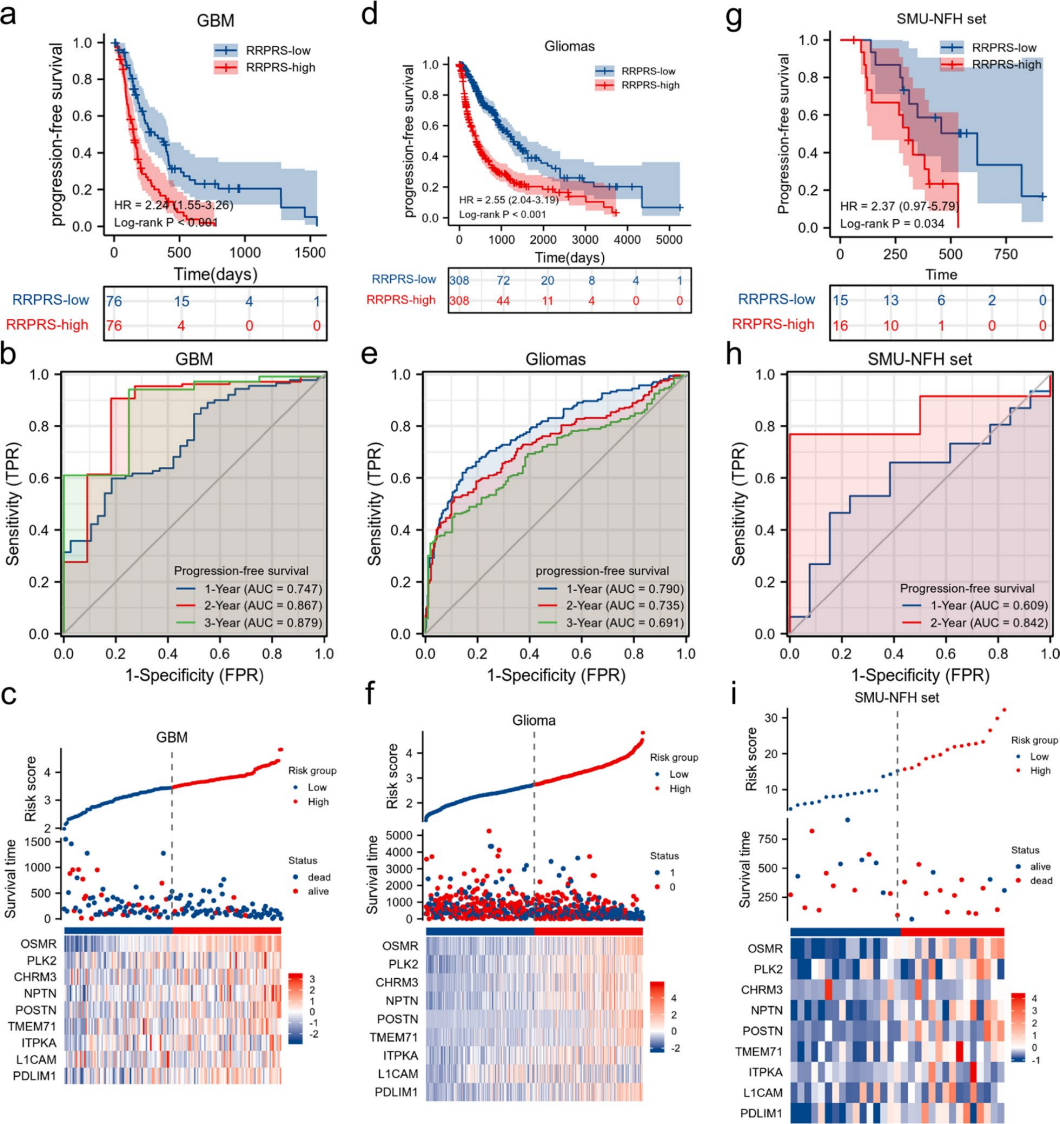
The predictive value of the RRPRS for the PFS of patients with glioma. Kaplan–Meier plots, time‐dependent ROC curves, the distributions of risk scores, survival status, and expression of radiosensitivity-related genes in the training set (**a**–**c**), validation set (**d**–**f**), and the SMU-NFH cohort (**g**–**i**). Significance for PFS analysis was calculated using a log-rank test, with the red line representing the RRPRS-high-risk group and the blue line representing the RRPRS-low-risk group. Red in the heatmap represents a high expression level and blue represents a low expression level

Using the median risk score as the cutoff value, the training set patients were divided into RRPRS-high-risk (n = 76) and RRPRS-low-risk groups (n = 76). The same approach was used for the validation set and test cohort. In the training set, the Kaplan–Meier survival curve showed that the PFS of the RRPRS-high-risk group was significantly shorter than that of the low-risk group (P < 0.001) (Fig. 3a). Furthermore, the model built using the RRPRS showed good performance on the ROC analysis for PFS, with AUC values of 0.747, 0.867, and 0.879 for 1-year, 2-year, and 3-year PFS, respectively (Fig. 3b). Figure 3c illustrates the risk score distribution, survival status distribution, and gene expression of each patient in the training set. The heat map showed that the high-risk group had higher expression levels of OSMR and PLK2, while the low-risk group had lower expression levels of ITPKA and L1CAM. In the validation set and SMU-NFH set, the PFS for the RRPRS-high-risk group was also shorter (P < 0.001 and P = 0.034, log-rank test) (Fig. 3d, g). The AUCs for 1-year, 2-year, and 3-year PFS were 0.778, 0.689, and 0.673 in the validation set, respectively, and 0.609 and 0.847 in the data from our center (Fig. 3e, h). The risk score, survival information, and the expression distributions of the nine genes in the patients are shown in Fig. 3f and i.

### Radiotherapy stratification analysis

**Fig. 4:**
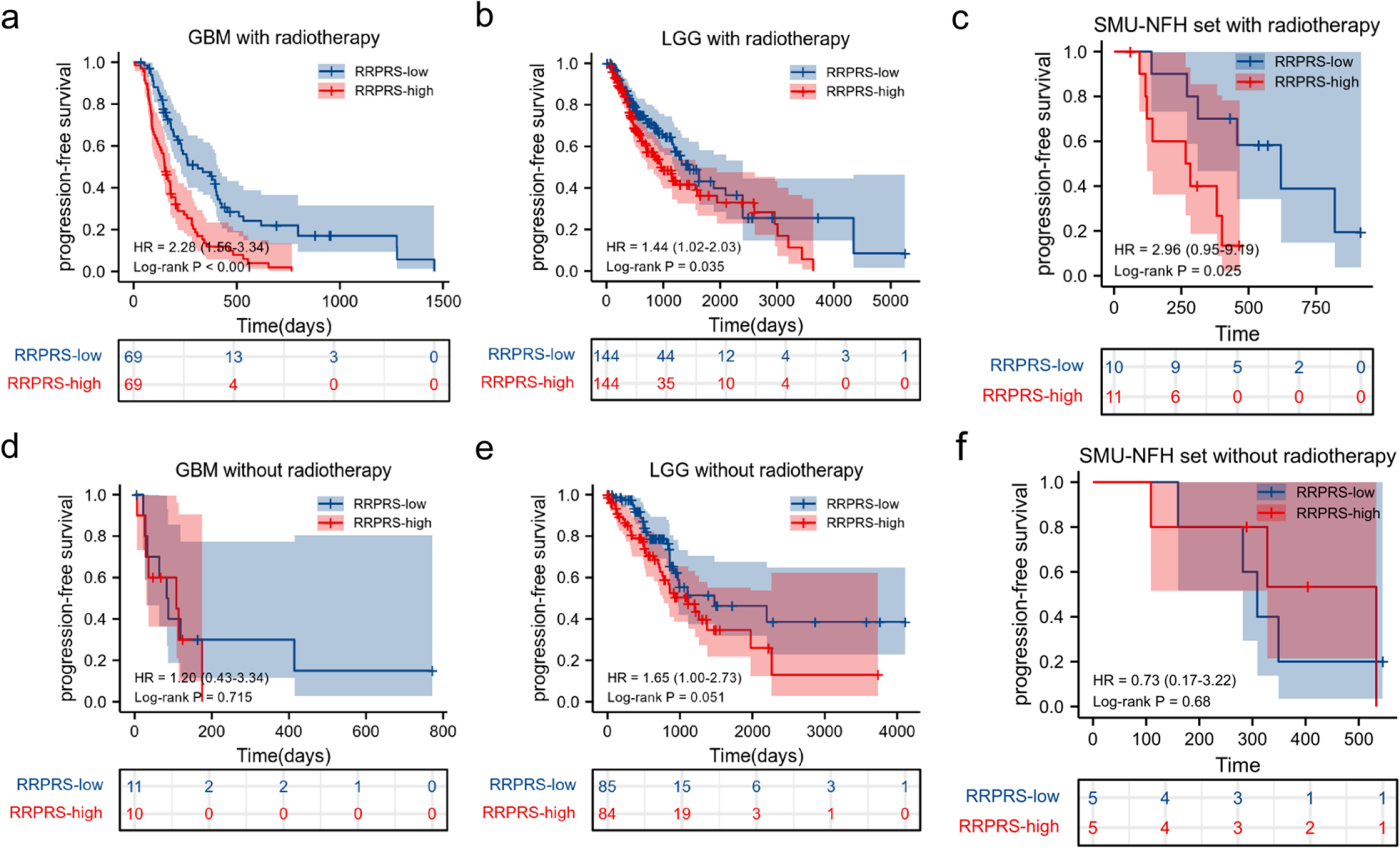
Radiotherapy stratification analysis. The RRPRS could further divide patients with glioma with radiotherapy (**a**–**c**) or without radiotherapy (**d**–**f**) into two groups with significantly different PFS values

To evaluate whether the RRPRS can predict radiation therapy outcomes in patients with glioma, we performed stratified analyses in the validation set and SMU-NFH set since most patients from the TCGA datasets had received radiotherapy according to the clinical information shown in Table 1. The PFS in the different risk groups of patients with glioma who received radiotherapy was statistically significant (Fig. 4a–c), in contrast to the non-radiotherapy group (Fig. 4d–f). To further verify the effectiveness of the predictor, the CGGA database was used to validate the prediction efficacy of RRPRS. Since the data on PFS of patients were lacking in the CGGA database, we used overall survival for analysis. The Kaplan–Meier curve analysis in patients who received radiotherapy revealed that those who had a high RRPRS-risk score had significantly worse overall survival (OS) compared to those with a low RRPRS-risk score (P = 0.02) (Additional file 1: Fig. S2a). In contrast, no significant difference was observed in the non-radiotherapy group (Additional file 1: Fig. S2b). Additional file 1: Fig. S2c showed the relationship between risk score distribution and nine-genes expression distribution of patients in the CGGA database. In summary, the results suggest that the RRPRS performed well in predicting the progression-free survival (PFS) in patients undergoing radiation therapy.

### Stratified PFS analysis based on the risk model in the validation set of the TCGA cohort

**Fig. 5:**
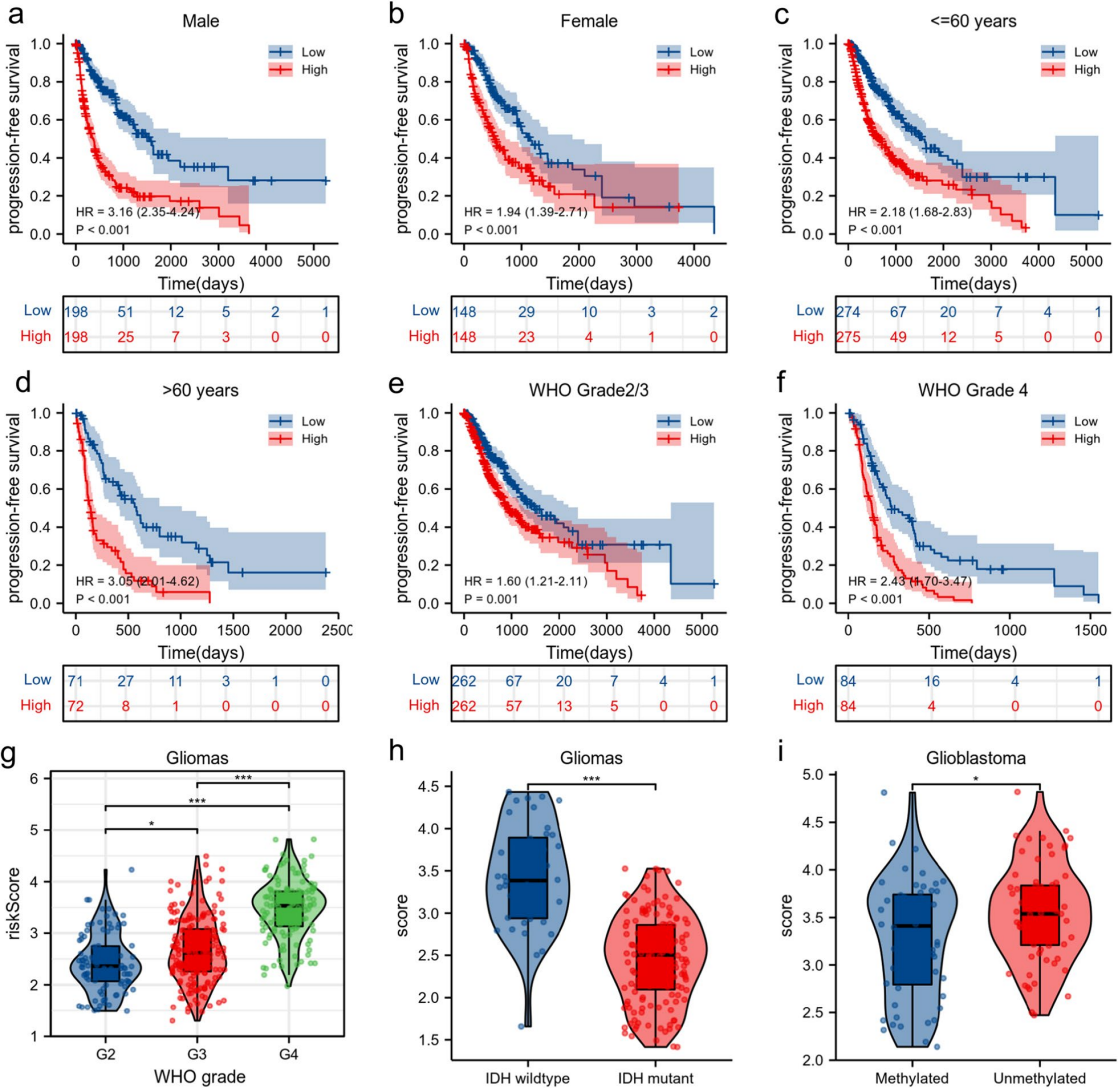
Stratified PFS analysis based on the risk model in the validation set of the TCGA cohort. Based on the RRPRS, stratified PFS analysis was performed in patients with glioma with different clinical parameters for the validation set, such as sex (**a**, **b**), age group (**c**, **d**), CNS WHO classification (**e**, **f**), and the relationship between the risk score and histological type (**g**), IDH mutation status (**h**), MGMT promoter status (**i**). Significance for survival analysis was calculated using Cox regression, with the red line representing the RRPRS-high-risk group and the blue line representing the low-risk group

To verify the predictive efficiency in stratified cohorts, patients were stratified according to other clinical parameters in the validation set, such as sex, age (≤ 60/> 60 years), and CNS WHO classification (Grade II/III/IV). Kaplan–Meier curves showed that the high-risk group had a shorter PFS than the low-risk group in all subgroups (P ≤ 0.001) (Fig. 5a–f), indicating that the RRPRS risk score is a robust predictor of PFS across all subgroups. We further explored the relationship between the risk score and clinical parameters in patients with glioma. Higher risk scores were associated with higher histopathological glioma grade (Fig. 5g). Additionally, IDH wildtype status and MGMT promoter unmethylated status demonstrated higher risk scores, indicating that the risk score is associated with resistance to chemoradiotherapy. Overall, our results showed that the RRPRS risk score is a reliable predictor of PFS in glioma patients.

### Survival analysis for the genes in the RRPRS

**Fig. 6:**
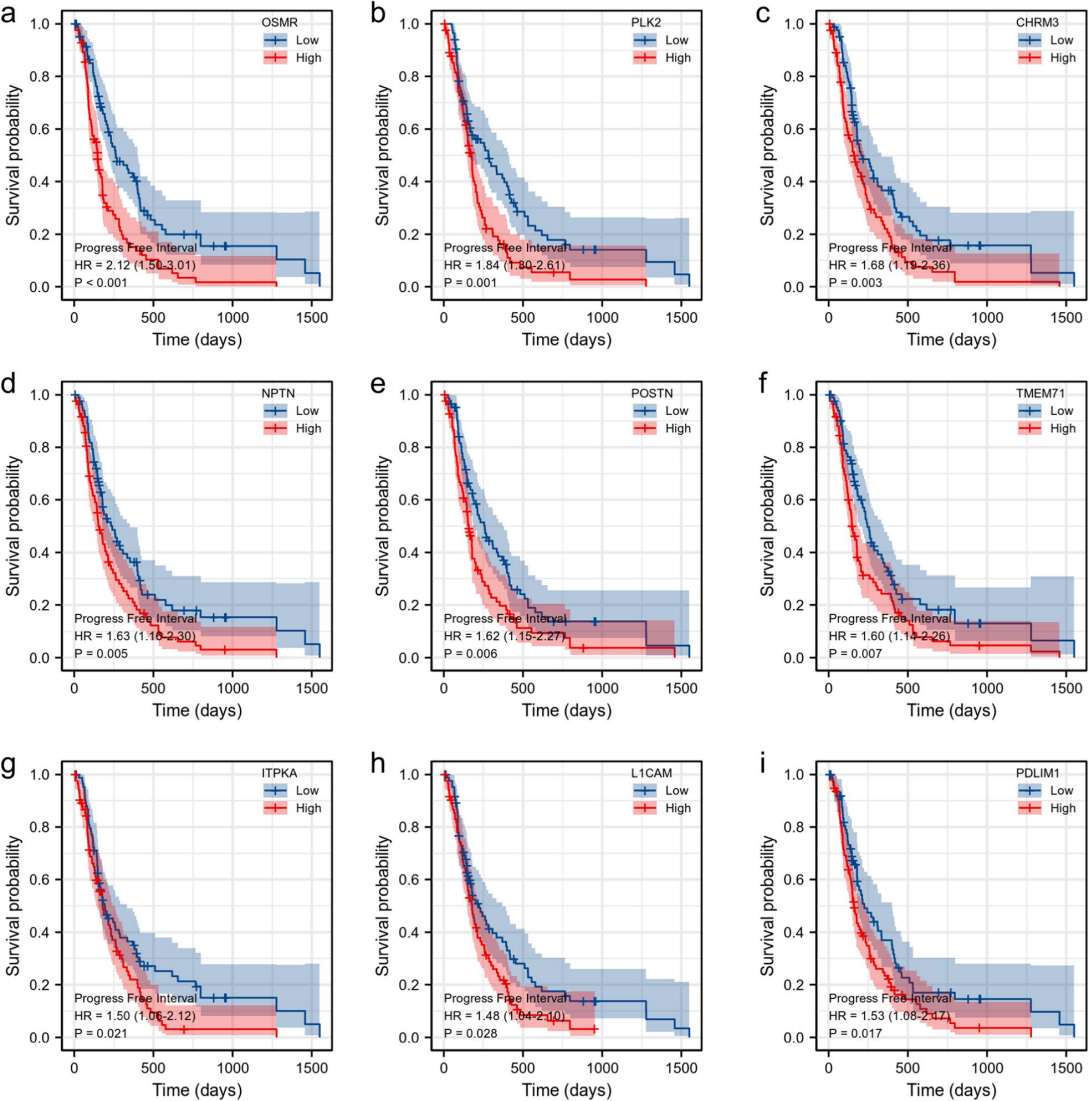
Survival analysis associated with the genes in Radiosensitivity-Related Prognostic Risk Signature (RRPRS). Kaplan–Meier plots with the PFS probability of the patients according to stratification by the expression levels of each individual gene. The ordinate axis represents the probability of survival (0–1), and the abscissa axis represents the PFS in days. Blue represents patients with gene expression levels below the median expression of the gene, and red represents patients with expression levels above the median

To determine the prognostic value of the nine genes in the RRPRS, we calculated the risk score for each patient with glioblastoma in the TCGA dataset and divided them into high-risk and low-risk groups based on the median risk score. We then performed a Kaplan–Meier survival analysis for each of the nine genes using the “survival and survminer package” in R (Fig. 6a–i). The results showed that these nine genes significantly affected the PFS of the patients, and most of the genes also impacted the OS of patients with GBM (Additional file 1: Fig. S3). The survival time of PFS and OS in the high-expression group was significantly lower than that in the low-expression group. However, there was no significant difference in OS between the high and low-expression groups for *NPTN* and *TMEM71*.

### Establishment of nomograms to predict the OS and PFS in patients with glioma

**Fig. 7:**
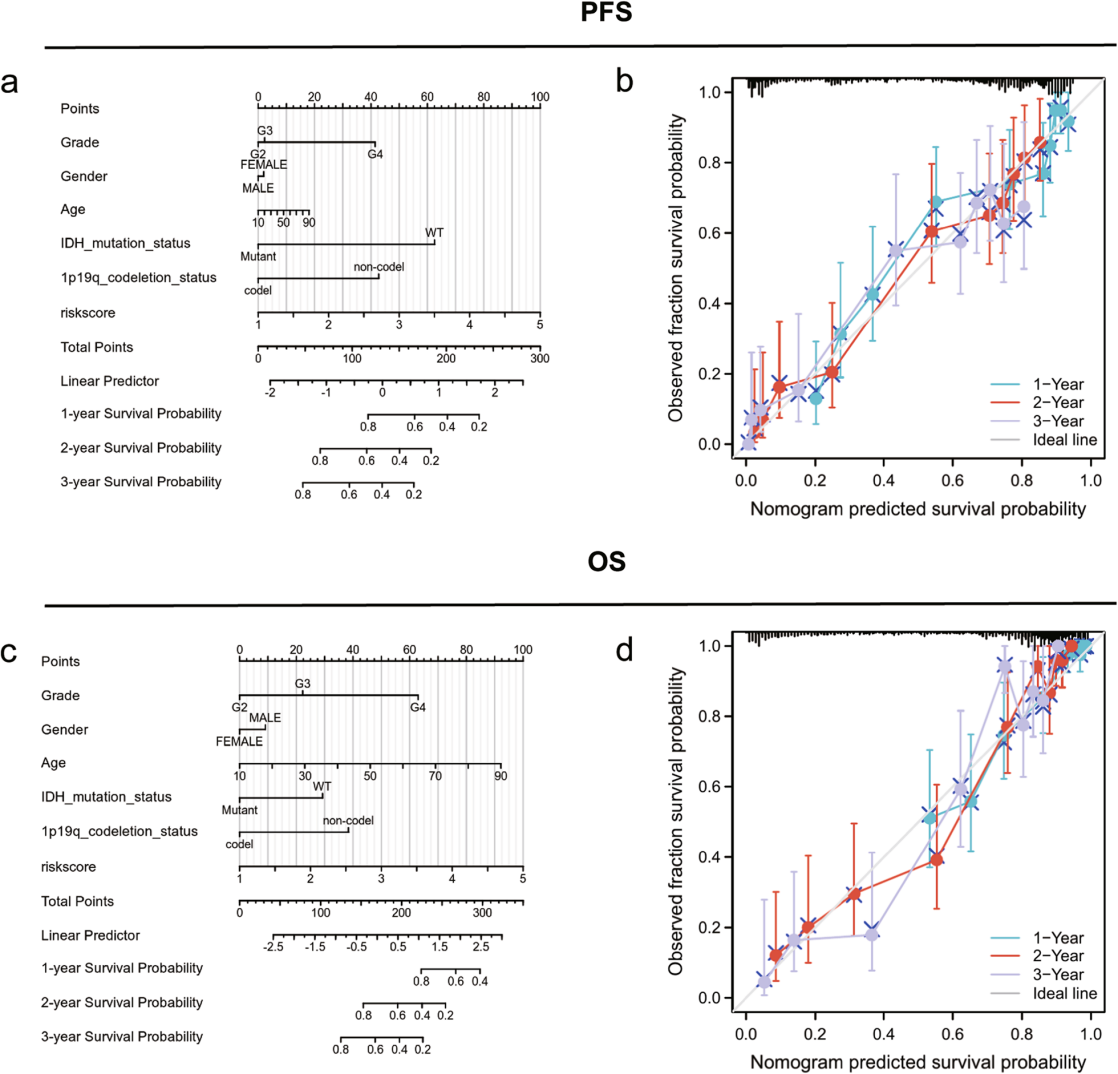
Establishment of nomograms to predict OS and PFS in patients with glioma. The nomogram **a**, **c** covering gender, age, Karnofsky physical status (KPS), IDH mutation status, 1p19q codeletion status and risk score, at 1-year, 2-year, and 3-year in patients with glioma. **b**, **d** calibration curve for the PFS and OS nomogram models

To further improve the predictive accuracy of the RRPRS, we established prognostic nomograms for PFS and OS, which included the risk score and other independent prognostic factors from the multivariate analysis. As shown in Fig. 7a and c, the higher the total score based on the sum of the assigned numbers for each factor in the nomograms, the worse the 1-year, 2-year, and 3-year PFS and OS rates. Subsequent analysis showed that the 1-, 2-, and 3-year PFS and OS calibration curves were close to the ideal curve, suggesting that the nomogram could accurately predict the radiation therapy outcomes of patients with glioma (Fig. 7b and d).

## Discussion

Radiotherapy is a standard treatment modality for high-grade gliomas, which could improve the OS survival time from months to years. However, with treatment prolongation, radiotherapy resistance commonly occurs in the radiation field of glioma patients. Despite the continuous improvement of radiotherapy techniques including Intensity Modulated Radiation Therapy (IMRT), Volumetric Modulated Arc Therapy (VMAT), and Tomotherapy (TOMO) in recent years aimed at making radiotherapy more individualized and precise, a large proportion of patients with high-grade glioma still experience recurrence after radiotherapy. Hence, identifying features related to radiotherapy sensitivity is still a significant challenge. Some investigators have discovered differentially expressed genes (DEGs) profiles and signatures in glioma by mining data from the GEO and TCGA databases. For example, Yan et al. [20] suggested that the prognosis of patients in the glioma radio-resistant group is significantly worse than that in the radio-sensitive group. Lin et al. [18] analyzed the lncRNA-expression profiles of 167 LGG patients from the TCGA database and developed a risk-score model based on the expression of 3 lncRNAs, which were significantly associated with OS in patients with glioma. Zhang et al. [19] established a five-microRNA signature according to the association of the LGG patients’ overall survival (OS) with microRNA expression. Recently, Yan et al. [20] analyzed genes significantly associated with OS in radiotherapy patients and intersected immune-related genes to construct a 21-gene signature to identify patients with LGGs who would benefit from radiotherapy. However, these studies mostly failed to control for confounding factors or provide justification for them, such as the extent of surgical resection, chemotherapy regimens, and compliance, which might lead to clinical response or survival information that cannot fully reflect radio-sensitivity. Therefore, the clinical application of prognostic biomarkers in glioma remains very limited to date. Here, we constructed glioma radio-sensitive and radio-resistant cell lines for microarray analysis and reported the first DEGs profiling related to the radiotherapy sensitivity of glioma.

In this study, we took the intersection of the DEGs obtained from the microarray analysis and the prognostic-related genes screened from the online databases, particularly those DEGs that correlated with PFS and treatment response. Subsequently, LASSO-COX regression analysis was used to establish a nine-gene prognostic signature related to radiotherapy sensitivity. Notably, we identified a novel 9 gene radio-sensitivity signature for patients with glioma in this cohort. Kaplan Meier survival analysis demonstrated the effectiveness of the 9 genes in predicting the response to radiotherapy treatment. Multivariate Cox-regression analysis identified the 9 genes signature as an independent prognostic factor for patients with glioma. We demonstrated that radiotherapy provides a clear survival benefit to patients classified as low-risk by the 9 genes The further use of this classifier signature might allow us better identification of patients who are likely to benefit from conventional radiotherapy. Therefore, our RRPRS model for patients with glioma serves as both a prognostic and a predictive approach, as patients in the high-risk group are more likely to relapse and less sensitive to radiation therapy. This underscores the importance of administering higher dose radiotherapy or other high-intensity treatments, including immunotherapy and targeted therapies, to high-risk patients as soon as possible.

The 9 genes RRPRS is composed of *OSMR*, *PLK2*, *CHRM3*, *NPTN*, *POSTN*, *TMEM71*, *ITPKA*, *L1CAM*, and *PDLIM1*. Although individual studies have previously investigated these genes, their collective impact on the response to radiotherapy remains unclear. Oncostatin M receptor (OSMR) belongs to the interleukin-6 receptor family. Ahmad et al. [29] found that OSMR could regulate glioma stem cell respiration and confer resistance to radiotherapy via the regulation of oxidative phosphorylation. Polo like kinase 2 (PLK2), a member of the serine/threonine kinases family, could induce glioblastoma resistance to temozolomide by regulating Hes family BHLH transcription factor 1 (HES1) and notch signaling [30]. Cholinergic receptor muscarinic 3 (CHRM3), a member of the G protein-coupled receptor family, was shown to inhibit tumor cell viability, colony formation, migration, and invasion, and promote apoptosis when silenced [31]. Neuroplastin (NPTN), which encodes a type I transmembrane protein belonging to the Ig superfamily, can promote lung cancer progression through epithelial-mesenchyme transition (EMT) [32]. Periostin (POSTN) was found to be directly related to glioma tumor grade and recurrence, promoting glioma cell invasion and adhesion while increasing the survival rate of glioma stem cells [33]. Previous studies [34] have found that the transmembrane protein 71 (TMEM71) is upregulated in GSCs and Temozolomide-resistant cells, and TMEM71 expression correlated positively with the degree of glioma malignancy, which might be mediated by involvement in tumor immune and inflammatory responses, cell proliferation, cell migration, chemotaxis, and response to drugs. Ma et al. [35] suggested that inositol-trisphosphate 3-kinase A (ITPKA) might promote transcriptional dysregulation through methylation, leading to glioma development and progression. L1 cell adhesion molecule (L1CAM) was shown to enhance the radiation resistance of glioma stem cells by increasing the phosphorylation of ATM and CHK2, slowing down cell death, and improving the formation efficiency and size of GSC tumor spheres [36]. PDZ and LIM domain 1 (PDLIM1) might be associated with glioma invasion and was highly expressed in multicentric GBM, suggesting its potential role in promoting GBM aggressiveness [37]. Although the specific mechanisms by which these genes affect sensitivity to radiotherapy require further study, the results suggested that the sensitivity and specificity of the RRPRS were significantly higher than for any single gene. There were significant survival differences between the high-and low-risk groups according to the RRPRS. The training and validation groups demonstrated the predictive power of RRPRS for the survival of patients with glioma.

During the enrollment of patients in our center, some patients did not receive radiotherapy because of financial or personal reasons, which also existed in the TCGA database. Therefore, we divided the population into a radiotherapy group and a non-radiotherapy group. We found that among patients who received radiotherapy, the incidence of death and disease progression in the low-risk patients was lower, and the high-risk group had a higher likelihood of disease progression and poor prognosis. There was no significant survival difference between the two groups that did not receive radiotherapy, suggesting that RRPRS is specific to radiotherapy and only predicts the outcome of radiotherapy patients. This also showed the significance of the prognostic model in the study of radiotherapy sensitivity, which might have a guiding significance to predict the recurrence time of radiotherapy patients. Therefore, during the process of clinical radiotherapy, those high-risk patients could receive radiotherapy earlier or a higher dose of the local tumor, with the hope of reducing the risk of recurrence and improving tumor control rates and survival. For low-risk patients, a relatively low radiotherapy dose could be given to reduce the possibility of radiation brain injury under the condition of ensuring lower recurrence risk and better survival time.

However, there are some limitations to this study. Firstly, the six samples of glioma radiation-sensitive and resistant cells were constructed in vitro for chip analysis. In this process, we only considered the difference in radiotherapy sensitivity caused by the simple tumor cells, ignoring the influence of the tumor microenvironment. Secondly, during the validation of our cohort, we only included a small sample size of 31 patients with glioblastoma, which may not be representative of all patients with glioma. Thirdly, our study, whether from a public database or our cohort, was retrospective in nature, and the results have not been validated in a prospective cohort. Finally, further exploration of the molecular mechanisms underlying radiotherapy resistance is required to identify potential therapeutic targets that can be translated into clinical practice.

## Conclusions

In this study, we used microarray analysis of the radio-sensitive and radio-resistant glioma cell lines (M059J and M059K) to explore their differential gene expression patterns. Using the intersection of the prognostic genes identified from the TCGA and GEO, we obtained several radio-sensitive related genes. Then, LASSO-COX regression analysis was used to establish prognosis characteristics related to radiotherapy sensitivity. Then, we validated the novel RRPRS in different cohorts to predict disease recurrence after radiotherapy for patients with glioma. The results showed that the RRPRS could be a prognostic indicator for patients with glioma, which could predict PFS and help to identify patients with glioma who are likely to benefit from radiotherapy. Our results indicated that the radiosensitivity-related nine-gene prognostic risk signature is potential and prognostic indicator for patients with glioma treated using radiotherapy.

### Supplementary Information

**Additional file 1: Figure S1.** Gene sets enriched in M059K and M059J cell lines, which were resistant and sensitive to radiotherapy, respectively (P < 0.05, false discovery rate < 0.25). Red represents the radio-resistant group and blue represents the radio-sensitive group. **Figure S2.** Radiotherapy stratification analysis in the CGGA. The RRPRS could further divide patients with glioma with radiotherapy (**a**) or without radiotherapy (**b**) into two groups with significantly different OS values. The distributions of risk scores and expression of radiosensitivity-related genes in the CGGA database (**c**). **Figure S3.** Survival analysis associated with the genes in Radiosensitivity-Related Prognostic Risk Signature (RRPRS). Kaplan–Meier plots with the OS probability of the patients according to stratification by the expression levels of each gene. The ordinate axis represents the probability of survival (0–1), and the abscissa axis represents the overall survival in days. Blue represents patients with gene expression levels below the median expression of the gene, and red represents patients with expression levels above the median. **Table S1.** Univariate and multivariable Cox regression analysis of OS in the CGGA. **Table S2.** Clinical and pathological data from 31 glioblastomas patients of the Nanfang Hospital, Southern Medical University (SMU-NFH) cohort.

## Data Availability

The data could be downloaded at (https://portal.gdc.cancer.gov/, https://xenabrowser.net/) and the code used during the current study are available from the corresponding author on reasonable request.

## Abbreviations

GBM: Glioblastoma
PFS: Progression-free survival
OS: Overall survival
DFS: Disease-free survival
DEGs: Differentially expressed genes
TCGA: The Cancer Genome Atlas
RRPRS: Radiosensitivity-related prognostic risk signature
mRNA: Message RNA
AUC: Area under the curve
GSCs: Glial stem cells
GSEA: Gene set enrichment analysis
KPS: Karnofsky physical status
