# Supplementary figures and images for: Exploration of the radiosensitivity-related prognostic risk signature in patients with glioma: evidence from microarray data
Source: J Transl Med. 2023 Sep 12;21:618. doi: 10.1186/s12967-023-04388-w (PMC10496232; doi:10.1186/s12967-023-04388-w)

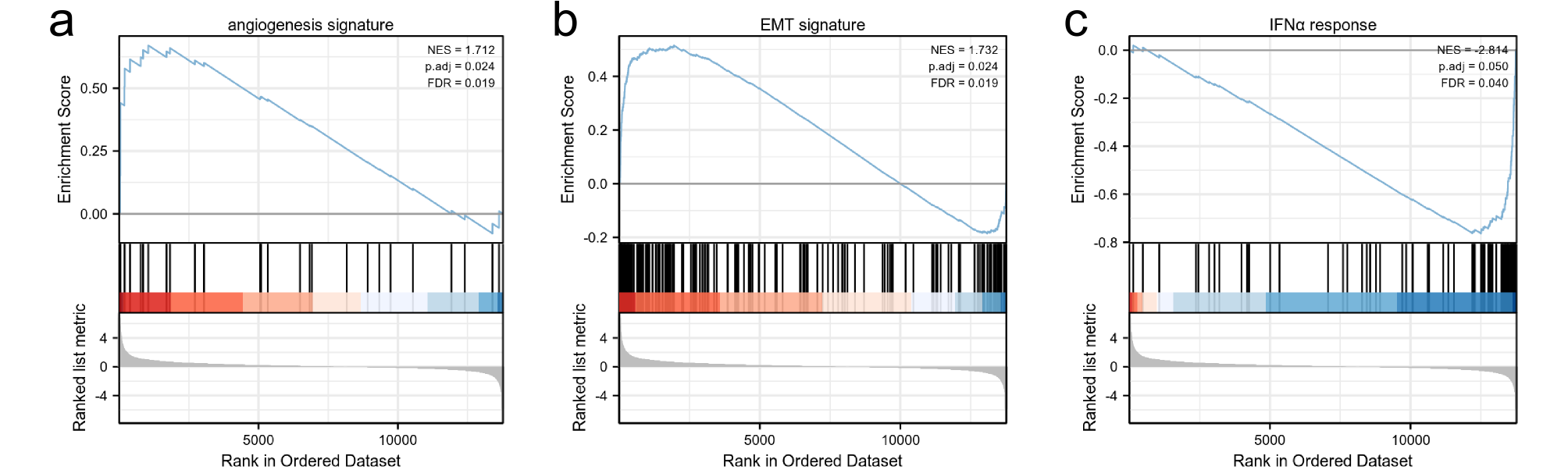

Supplement: Supplementary file 1 — Additional file 1: Figure S1. Gene sets enriched in M059K and M059J cell lines, which were resistant and sensitive to radiotherapy, respectively (P < 0.05, false discovery rate < 0.25). Red represents the radio-resistant group and blue represents the radio-sensitive group. Figure S2. Radiotherapy stratification analysis in the CGGA. The RRPRS could further divide patients with glioma with radiotherapy (a) or without radiotherapy (b) into two groups with significantly different OS values. The distributions of risk scores and expression of radiosensitivity-related genes in the CGGA database (c). Figure S3. Survival analysis associated with the genes in Radiosensitivity-Related Prognostic Risk Signature (RRPRS). Kaplan–Meier plots with the OS probability of the patients according to stratification by the expression levels of each gene. The ordinate axis represents the probability of survival (0–1), and the abscissa axis represents the overall survival in days. Blue represents patients with gene expression levels below the median expression of the gene, and red represents patients with expression levels above the median. Table S1. Univariate and multivariable Cox regression analysis of OS in the CGGA. Table S2. Clinical and pathological data from 31 glioblastomas patients of the Nanfang Hospital, Southern Medical University (SMU-NFH) cohort. [file 12967_2023_4388_MOESM1_ESM.zip › Additional file 1/Fig S1.tif]

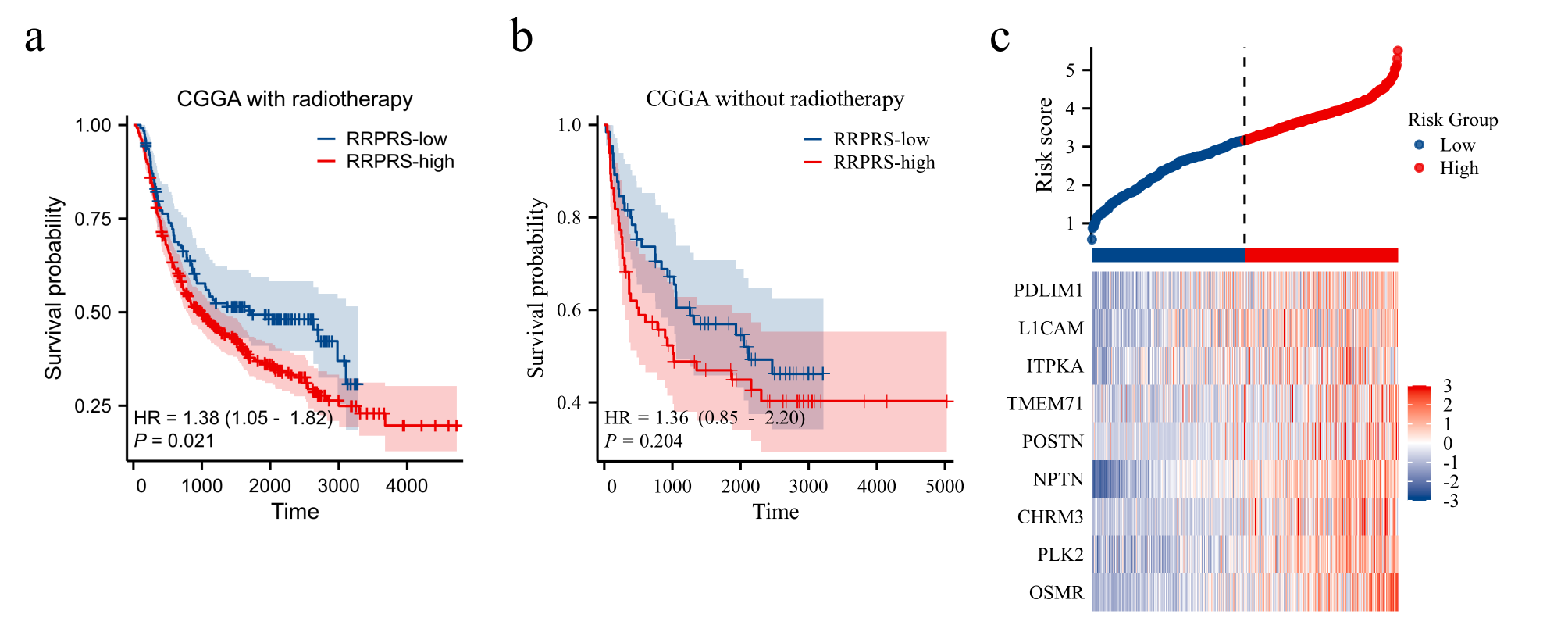

Supplement: Supplementary file 1 — Additional file 1: Figure S1. Gene sets enriched in M059K and M059J cell lines, which were resistant and sensitive to radiotherapy, respectively (P < 0.05, false discovery rate < 0.25). Red represents the radio-resistant group and blue represents the radio-sensitive group. Figure S2. Radiotherapy stratification analysis in the CGGA. The RRPRS could further divide patients with glioma with radiotherapy (a) or without radiotherapy (b) into two groups with significantly different OS values. The distributions of risk scores and expression of radiosensitivity-related genes in the CGGA database (c). Figure S3. Survival analysis associated with the genes in Radiosensitivity-Related Prognostic Risk Signature (RRPRS). Kaplan–Meier plots with the OS probability of the patients according to stratification by the expression levels of each gene. The ordinate axis represents the probability of survival (0–1), and the abscissa axis represents the overall survival in days. Blue represents patients with gene expression levels below the median expression of the gene, and red represents patients with expression levels above the median. Table S1. Univariate and multivariable Cox regression analysis of OS in the CGGA. Table S2. Clinical and pathological data from 31 glioblastomas patients of the Nanfang Hospital, Southern Medical University (SMU-NFH) cohort. [file 12967_2023_4388_MOESM1_ESM.zip › Additional file 1/Fig S2.tif]

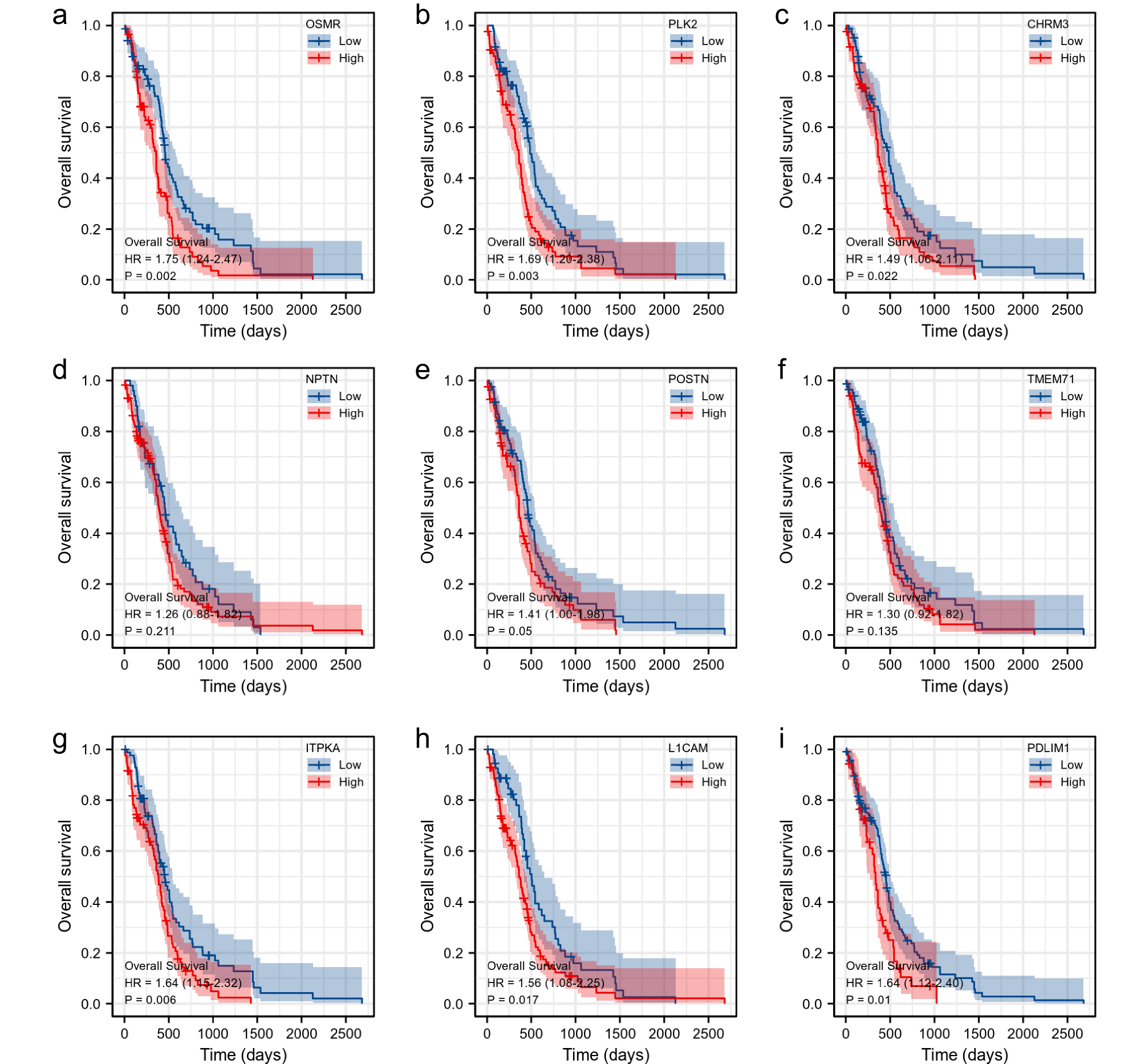

Supplement: Supplementary file 1 — Additional file 1: Figure S1. Gene sets enriched in M059K and M059J cell lines, which were resistant and sensitive to radiotherapy, respectively (P < 0.05, false discovery rate < 0.25). Red represents the radio-resistant group and blue represents the radio-sensitive group. Figure S2. Radiotherapy stratification analysis in the CGGA. The RRPRS could further divide patients with glioma with radiotherapy (a) or without radiotherapy (b) into two groups with significantly different OS values. The distributions of risk scores and expression of radiosensitivity-related genes in the CGGA database (c). Figure S3. Survival analysis associated with the genes in Radiosensitivity-Related Prognostic Risk Signature (RRPRS). Kaplan–Meier plots with the OS probability of the patients according to stratification by the expression levels of each gene. The ordinate axis represents the probability of survival (0–1), and the abscissa axis represents the overall survival in days. Blue represents patients with gene expression levels below the median expression of the gene, and red represents patients with expression levels above the median. Table S1. Univariate and multivariable Cox regression analysis of OS in the CGGA. Table S2. Clinical and pathological data from 31 glioblastomas patients of the Nanfang Hospital, Southern Medical University (SMU-NFH) cohort. [file 12967_2023_4388_MOESM1_ESM.zip › Additional file 1/Fig S3.tif]
